# Real-Time Investigation of Tuberculosis Transmission: Developing the Respiratory Aerosol Sampling Chamber (RASC)

**DOI:** 10.1371/journal.pone.0146658

**Published:** 2016-01-25

**Authors:** Robin Wood, Carl Morrow, Clifton E. Barry, Wayne A. Bryden, Charles J. Call, Anthony J. Hickey, Charles E. Rodes, Thomas J. Scriba, Jonathan Blackburn, Chacha Issarow, Nicola Mulder, Jeremy Woodward, Atica Moosa, Vinayak Singh, Valerie Mizrahi, Digby F. Warner

**Affiliations:** 1 Institute of Infectious Disease and Molecular Medicine (IDM), Faculty of Health Sciences, University of Cape Town, Cape Town, South Africa; 2 Desmond Tutu HIV Centre, IDM, University of Cape Town, Cape Town, South Africa; 3 Laboratory of Clinical Infectious Diseases, National Institute of Allergy and Infectious Diseases, US National Institutes of Health, Bethesda, Maryland, United States of America; 4 Zeteo Tech LLC, Ellicott City, Maryland, United States of America; 5 RTI International, Research Triangle Park, North Carolina, United States of America; 6 Aerosol Exposure Dimensions, Cary, North Carolina, United States of America; 7 South African Tuberculosis Vaccine Initiative, Department of Paediatrics and Child Health, University of Cape Town, Cape Town, South Africa; 8 Structural Biology Research Unit, Department of Integrative Biomedical Sciences, Faculty of Health Sciences, University of Cape Town, Cape Town, South Africa; 9 MRC/NHLS/UCT Molecular Mycobacteriology Research Unit & DST/NRF Centre of Excellence for Biomedical TB Research, Department of Pathology, Faculty of Health Sciences, University of Cape Town, Cape Town, South Africa; Fundació Institut d’Investigació en Ciències de la Salut Germans Trias i Pujol, Universitat Autònoma de Barcelona, SPAIN

## Abstract

Knowledge of the airborne nature of respiratory disease transmission owes much to the pioneering experiments of Wells and Riley over half a century ago. However, the mechanical, physiological, and immunopathological processes which drive the production of infectious aerosols by a diseased host remain poorly understood. Similarly, very little is known about the specific physiological, metabolic and morphological adaptations which enable pathogens such as *Mycobacterium tuberculosis* (*Mtb*) to exit the infected host, survive exposure to the external environment during airborne carriage, and adopt a form that is able to enter the respiratory tract of a new host, avoiding innate immune and physical defenses to establish a nascent infection. As a first step towards addressing these fundamental knowledge gaps which are central to any efforts to interrupt disease transmission, we developed and characterized a small personal clean room comprising an array of sampling devices which enable isolation and representative sampling of airborne particles and organic matter from tuberculosis (TB) patients. The complete unit, termed the Respiratory Aerosol Sampling Chamber (RASC), is instrumented to provide real-time information about the particulate output of a single patient, and to capture samples via a suite of particulate impingers, impactors and filters. Applying the RASC in a clinical setting, we demonstrate that a combination of molecular and microbiological assays, as well as imaging by fluorescence and scanning electron microscopy, can be applied to investigate the identity, viability, and morphology of isolated aerosolized particles. Importantly, from a preliminary panel of active TB patients, we observed the real-time production of large numbers of airborne particles including *Mtb*, as confirmed by microbiological culture and polymerase chain reaction (PCR) genotyping. Moreover, direct imaging of captured samples revealed the presence of multiple rod-like *Mtb* organisms whose physical dimensions suggested the capacity for travel deep into the alveolar spaces of the human lung.

## Introduction

In pioneering experiments over half a century ago, Wells and Riley confirmed the airborne transmission of *Mycobacterium tuberculosis* (*Mtb*) [1,2]. Moreover, Wells proposed the concept of an infectious quantum, a unit of infection capable of causing disease in 68.3% of susceptible animals [1]. Key conclusions of these experiments—in particular, that there are low numbers of infectious quanta and transmission is driven by a small subset of smear-positive patients—have remained largely unchallenged. Subsequent data from similar animal studies appear to reinforce these findings [3,4], although the experimental formats may have been characterized by even lower sensitivities than the initial work of Wells and Riley owing to the need for higher levels of diluting ventilation in modern health facilities [5]. In contrast, advances in molecular technology have enabled the detection of large numbers of *Mtb* DNA copies circulating in the air of TB treatment facilities [6,7,8]. Moreover, recent research [9] examining the exposure potentials of healthcare workers in a South African TB clinic demonstrated the utility of personal exposure sampling followed by PCR analysis as an effective tool to determine the risk of *Mtb* aerosol exposure. Importantly, these observations implicated the close proximity of health workers to patients—which resulted in concentrated exposure over shorter distances—as a possible reason for the inability of fixed-location aerosol sampling to detect aerosols in the same setting. Furthermore, the readily measureable breathing zone exposure described in this study strongly supports more extensive efforts to characterize the nature and sources of *Mtb* exposure and air transmission routes in different environments. Such knowledge is critical to inform the most robust mitigation recommendations for both occupational and residential settings; however it is heavily reliant on the development of practical tools to enable a more thorough understanding of the factors which determine *Mtb* particle production and transmission.

In clinical studies, there is a continued reliance on conventional bacteriology of sputum samples as a surrogate for infectiousness. Notwithstanding the practical benefits associated with a population that is easily sampled and analyzed, the dependence on sputum obscures critical uncertainties about the factors which ensure successful transmission of *Mtb* bacilli from one individual to another. These include the number of infectious particles produced, the number of bacilli within an infectious particle, the anatomical origin and composition of infective droplets, and the physiological adaptations which enable airborne transmission and viability of *Mtb*. Critically, for a disease which is driven by the host immune response [10], there is also incomplete knowledge about the mechanical, physiological, and immunopathological processes which underlie the production of infectious aerosols. Addressing these questions is not a simple undertaking, and requires the ability to observe in real time the release of *Mtb* particles by an infected patient, and to integrate this information within a complex dataset describing the host, bacterial, and environmental parameters which contribute to successful transfer of infecting bacilli to a new recipient. In other words, developing a systems biology of TB transmission.

Towards this end, we designed a small personal clean room in which airborne *Mtb* could be isolated in a clinical setting. Here, we detail the development and characterization of this Respiratory Aerosol Sampling Chamber (RASC), and present initial data suggesting its potential to accommodate newly diagnosed TB cases for enumeration and morphological characterization of *Mtb* particles. Our ultimate goal is to utilize this information to generate models predicting risks of TB exposure in specific environmental conditions, and to apply these models in developing practical interventions—clinical, operational, and infrastructural—to curb TB transmission in a region characterized by an elevated force of infection [11].

## Materials and Methods

### Ethics Statement

Approval for the human aspects of this research was obtained from the University of Cape Town Faculty of Health Sciences Human Research Ethics Committee (HREC/REF: 680/2013). Participants provided written informed consent prior to acceptance into the study.

### Experimental Description of RASC

**Fig 1:**
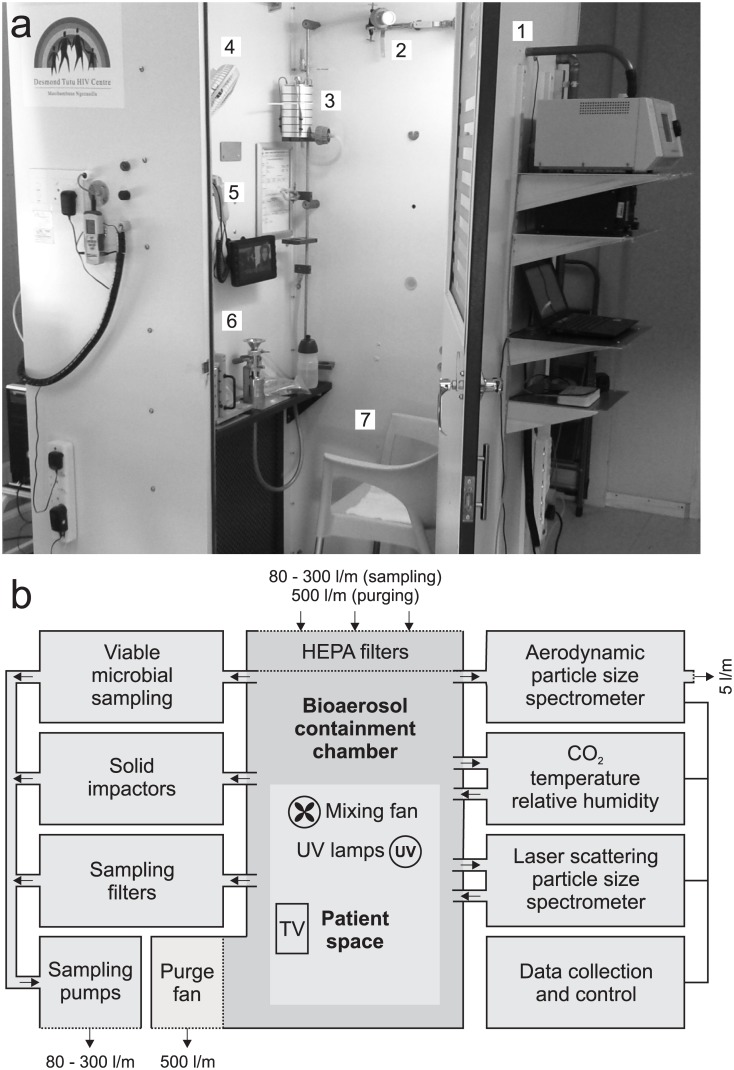
The Respiratory Aerosol Sampling Chamber (RASC). (A) Photograph of the RASC (with the door open) on site in a community TB clinic (1) aerodynamic particle sizer (2) Filter samplers (3) Andersen impactor (4) Mixing fan (5) CO2, temperature and RH (6) PM10 impactor (7) Chair for participant. (B) Block diagram depicting the fluidic and electronic configuration of the RASC. Thick connecting lines indicate airflow and aerosol paths; thin lines indicate electronic connections. All air leaving the RASC is HEPA filtered.

The RASC (Fig 1) developed for this study utilized the basic structure of a sputum collection booth (VividAir Cape Town, South Africa) which was modified to function as a small, personal cleanroom with a volume of 1.4 m^3^, enabling the quantitation and characterization of aerosol particles emitted by a seated study participant during normal breathing, coughing, and talking. The RASC was positioned in a community clinic, within a well-ventilated room undergoing 12 air changes per hour (ACH) of N95-filtered cross-flow ventilation. Attending staff were required to wear N95 respiratory masks throughout the experimental course.

**Fig 2:**
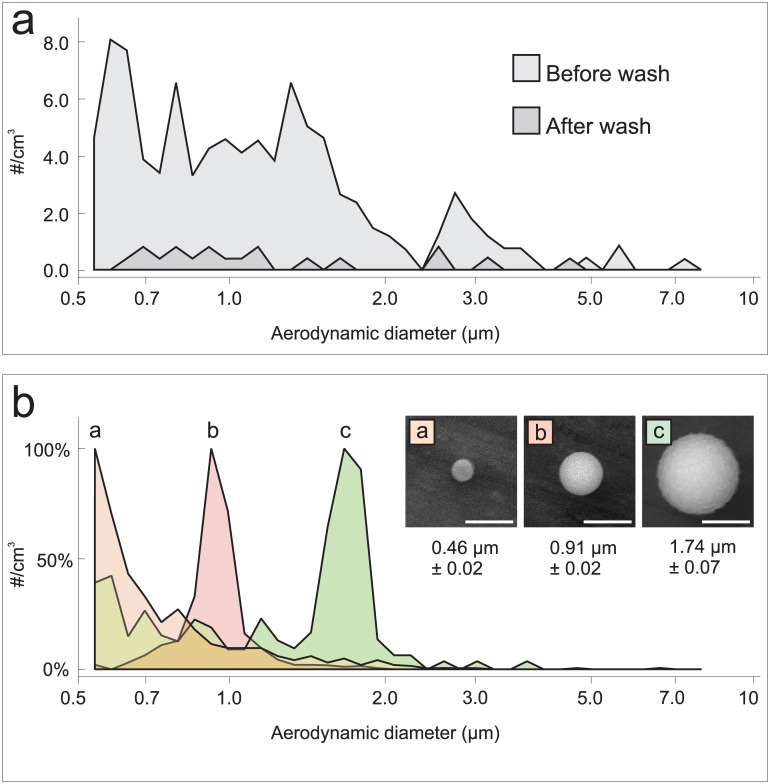
The in-built APS characterizes the particle size distribution spectrum within the RASC. (A) Typical background particle spectrum before and after air wash. Note the 10-fold decrease in particle counts across all size ranges following the air wash. Total count from a typical five seconds of sampling. (B) Artificial dry release of fluorescent polystyrene latex (PSL) microspheres. The APS instrument groups all particles with an aerodynamic diameter less than 0.523 μm in the number bin on the far left of each chart. Note that the 1μm release (b) contained approximately ten times more particles in the release. Total count from ten seconds of sampling at the peak of particle concentration. (Inset) corresponding SEM images of the released particles.

During prolonged sampling procedures, study participants were able to use a small television and entertainment system incorporated into the RASC. The RASC was hermetically sealed, and all incoming air was HEPA filtered. The effects of HEPA-filtered air washes on the background particle counts are shown in Fig 2A. The total particle count before the air wash was typically around 300 particles/cm^3^. After air wash, and prior to patient sampling, the integrated total background particle counts were generally fewer than 30 particles/cm^3^.

Study participants were required to wear full-body DuPont Tyvek suits throughout sampling procedures in order to minimize background particle clutter. The sealing door, incorporated a glass window for observation, could be opened externally as well as by subjects from inside the device. A small electric fan maintained internal air circulation (mixing), and temperature, humidity, and carbon dioxide levels were continuously monitored (Thermo-Hygro-CO_2_ monitor, MIC Meter Industry Co, Taiching, Taiwan). In the absence of air sampling or purging, the ventilation was determined as less than 0.1 ACH, and air-sampling rates during study protocols varied between 50–200 liters per minute (l/min). The sampled air being removed from the RASC was replaced by HEPA filtered air while the discharge after the sampling systems was HEPA filtered and released into the room. Carbon dioxide, a natural tracer gas produced during normal respiration, was used to quantify the volume of expired air that was sampled during each procedure. A high-volume exhaust system enabled purging of the booth at 500 l/min via a high-flow HEPA filter which was used prior to the patient’s exit from the RASC at the end of each study. Surface cleaning and inbuilt ultra-violet light irradiation were used to ensure equipment and surface sterilization, and to reduce cross contamination between studies.

### Bioaerosol Characterization

The RASC was equipped with a suite of continuously operating particle sensors to characterize aerosols with a temporal response of a few seconds. Central to the real-time measurement of aerosol particle concentration and particle size distribution was an Aerodynamic Particle Sizer (APS Model 3321, TSI, Shoreview, MN USA) and a Laser Scattering Particle Counter (Model 3016, Lighthouse Worldwide Solutions, Medford, Oregon USA). These two instruments worked in concert to characterize the aerosol particle environment in the RASC. The APS provided high-resolution aerodynamic (as opposed to physical) particle size distributions in the particle size range 0.7 μm to 20 μm. We assumed, based upon known deposition sites in the distal alveoli of the lungs, that the modal size of *Mtb*-infected sputum droplets emitted by patients would be centered within this range. Therefore, the measure of aerodynamic sizing was expected to mimic the way aerosols are sized and transported through the human respiratory system. The laser scattering particle counter provided lower resolution measurement of physical particle size but was particularly useful for the study of submicron-sized particles with a minimum size of 0.3 μm. In general, the information obtained from the APS was considered more likely to be relevant to the predicted behavior of particles in the lungs; however, the correlation between measurements on the two instruments (APS and laser scattering particle counter) provided rich data on the bio-aerosols generated by the patient during occupation of the RASC.

### Particle Sampling System

Our longer term goal is to utilize the RASC to define the morphological and molecular characteristics that might be key to transport of particles in the respiratory system and, in turn, determine the infectiveness of the pathogen. To this end, three main types of sample collectors were installed in the RASC, specifically: liquid impingers, physical impactors, and filters.

The liquid impingers (BioSampler, SKC, Eighty Four, PA) were set to sample at 12.5 l/min into liquid medium (PBS). Liquid impaction was included in the design of the RASC in order to enable the future use of FACS-based sorting of aerosolized *Mtb* bacilli from patients for downstream genotypic, phenotypic and infectivity characterizations, while solid impaction should additionally provide samples for whole-genome sequencing and mass spectrometry imaging to characterize the composition of airborne material collected on the impaction plates [12].

Two types of physical impactors were employed. A Dekati three-stage impactor (PM10 Impactor, Dekati, Kangasala, Finland) was set to sample at 30 l/min. The Dekati impactor was selected since it collects particles in three size ranges: for particles that have a density of 1 g.cm^-3^, the first stage collects particles >14.1 μm, the second stage collects particles in the 14.1 μm to 3.5 μm range, and the final stage collects particles in the 3.5 μm to 1.4 μm range. Therefore, this impactor was considered especially useful for imaging respired aerosol matter. A Six-stage Viable Andersen Cascade Impactor (Model 10830-EPD, Thermo Scientific, USA) was also employed since this device measures culturable particle counts as a function of particle size: each of the six stages captures smaller particles [13]. The airflow rate of the sampler was 28.3 l/min and the impaction plates comprised a thick coating of agar growth media to minimize bounce and enable microbial growth. Each of the impactor samplers was backed with a 0.4 μm filter to collect any particles that bypassed the impaction stages.

We also used two types of filter media to capture samples: micro-porous polycarbonate membranes and gel filters. The polycarbonate membrane filters (Sterlitech Corporation, WA USA) were nominally rated at 0.4 μm and provided high collection efficiencies when used in 47 mm open-faced filter holders, with flow rates through the filters usually around 28 l/min. The gel filters (Model 12602-37-ALK, Sartorius, Goettingen, Germany) were 37 mm in diameter, and rated to capture >99.99% of both bacterial and viral particles. These filters were selected since the gel-filtering medium maintains moisture so that collected organisms can maintain significant viability. Furthermore, the gel medium is soluble in water enabling high levels of organism recovery from the filters. The filters, which sample at up to 20 l/min, were used for shorter periods of time during the experiments to reduce dehydration and increase the likelihood of capturing viable organisms.

### Bacteriological, Molecular and Imaging Analyses

The *M*. *smegmatis*::*gfp* reporter mutant expressing green fluorescent protein (GFP) was constructed by introduction of the pMSP12GFP plasmid [14] into *M*. *smegmatis* mc^2^155. For determination of CFU in controlled aerosol release experiments, bacilli were propagated in liquid 7H9 medium (Difco) supplemented with 0.05% Tween-80, 0.2% glycerol and albumin/NaCl/ glucose (ADC) complex and/or isolated on solid 7H10 agar (Difco) supplemented with 0.2% glycerol and oleic acid/albumin/dextrose/catalase (OADC) complex. Owing to the selectivity provided by the *aph* cassette on pMSP12GFP, all media contained kanamycin (Sigma) at a final concentration of 20 μg/mL. For the pilot TB studies, the Andersen impactor contained solid 7H10 agar (Difco) plates supplemented with 0.2% glycerol and oleic acid/albumin/dextrose/catalase (OADC) complex.

PCR genotyping of the *Mtb* RD9 region was performed by amplifying genomic DNA from putative Mtb CFU using the primer set RD9/qRTF (5’-tgagtggcgatggtcaacac-3’) and RD9/qRTR (5’-gatggcgttcggaaagaaac-3’).

For analysis by scanning electron microscopy (SEM), samples were air-dried on aluminium foil and imaged without coating using a Zeiss/Leo 1450 Scanning Electron Microscope equipped with a lanthanum hexaboride (LaB6) cathode. Images were captured in secondary electron mode at 10 kV.

## Results

### Aerosol characterization of the RASC

In advance of patient testing in the RASC, it was important to characterize the behavior of particles in the chamber and demonstrate that accurate results could be obtained from both artificial and live aerosol releases. For artificial release, fluorescent polystyrene latex (PSL) microspheres (Sigma-Aldrich Corp, St. Louis, MO and Polysciences Inc., Warrington, PA) were “dry” aerosolized into the chamber. The resulting size distribution graphs (Fig 2B) confirmed accurate calibration of the APS system for the three bead sizes: 0.5 μm, 1.0 μm, and 2.0 μm. Quantitative SEM methodology (Fig 2B) indicates that the actual bead sizes were 0.47 μm, 0.91 μm and 1.74 μm. After PSL beads were introduced into the RASC, they were observed by the APS within 6 seconds and were well mixed within 20 seconds of release. Analysis of the APS size-bins associated with the 2 μm beads indicated that the count of PSL beads relative to background clutter prior to release was approximately 50. This ratio was defined as the “signal to clutter ratio” and, for the 1 μm beads, the ratio was calculated as approximately 250.

**Fig 3:**
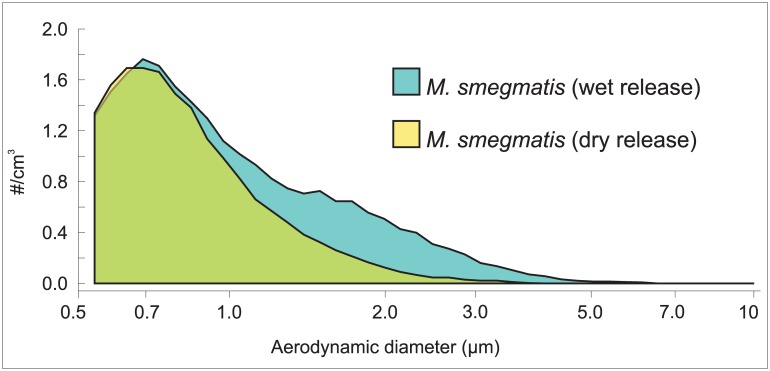
The effect of aerosol hydration on particle size distribution. The graph shows APS measurements of “wet” and “dry” releases of *M*. *smegmatis*::*gfp*. (see text for details).

For live aerosol release, we utilized a fluorescent reporter mutant of *M*. *smegmatis* mc^2^155, since the non-pathogenic mycobacterial model organism is of equivalent size and morphology to *M*. *tuberculosis* [15]. Suspensions of the exponentially growing *M*. *smegmatis*::*gfp* reporter strain were aerosolized into the RASC at several different concentrations to test the capacity of the system to characterize bioaerosols of mycobacteria accurately. Two different protocols were used to aerosolize *M*. *smegmatis*::*gfp*: an adapted micro paint sprayer that was fed by high pressure HEPA-filtered air to create a “wet” aerosol with heavy particle clustering; and an ultrasonic, vibrating orifice nebulizer (Model DPD-1b, BioProcess Diagnostics, Albuquerque, NM) that fed aerosol through a heated tube to create a “dry” aerosol. The aerosol was considered “dry” if its aerodynamic size distribution was unchanged over time scales of 10–20 s after exiting the drying tube. The two different release protocols had differential effects on the particle size distribution (Fig 3). Wet release skewed the particle size distribution to larger particles with a secondary maximum at a cluster size near 2 μm. In contrast, the dry release resulted in a particle size distribution with a single maximum near 0.8 μm, which was indicative of much smaller amounts of agglomeration and clustering.

We also used chalk dust (Visolite Tracer Compound PN 100–0164, BHA Group, Inc., Kansas City, MO) to characterize the loss of particles over time owing to surface deposition as a function of particle size (data not shown). Following release of chalk dust into the RASC, the peak was observed at 1 μm, but there were significant numbers of particles up to 10 μm in size. In 6 minutes following dust aerosolization, both total particle counts and the 1 μm particle counts decreased at a rate of approximately 4% per minute. Slightly less than 10% of this loss could be attributed to the APS sampling, which permanently removed particles from the chamber at a rate of 5 litres per minute. The 5 μm particle loss rate was 9% per minute.

### Capture of viable organisms: the Andersen Cascade Impactor

**Fig 4:**
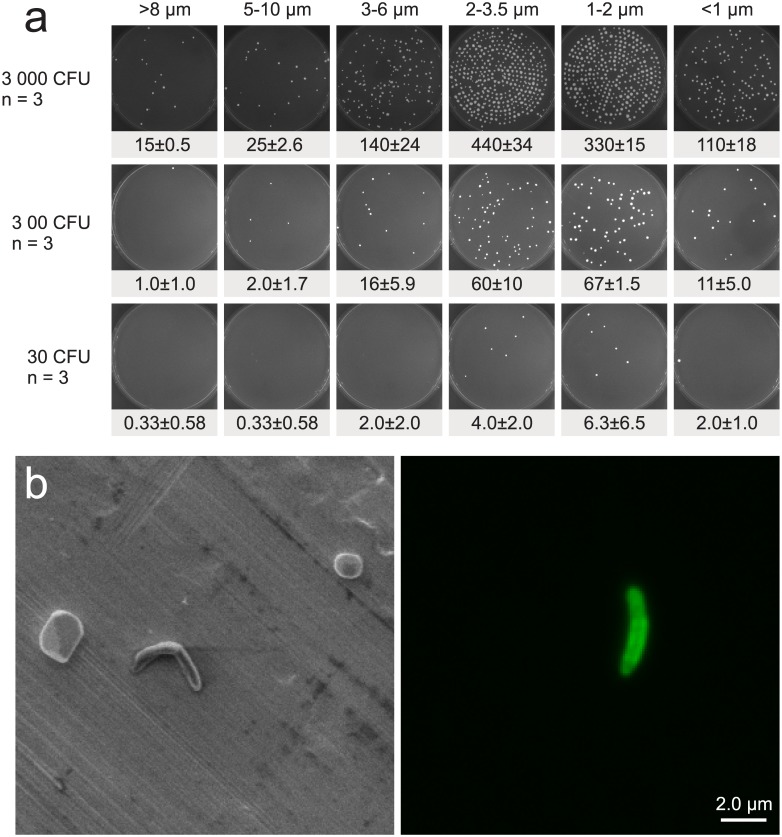
Isolation and visualization of viable mycobacteria in the RASC. (A) *M*. *smegmatis*::*gfp* growth on solid 7H10 agar plates from the Six-Stage Viable Andersen Cascade Impactor after wet release of 200 μl diluted culture into the RASC (30 000, 3000 and 300 colony forming units—CFU). The columns indicate the particle sizes captured on each plate across the 6 stages of the impactor, and the rows indicate the estimated total number of CFU passing through the impactor. Each release was repeated three times and the mean and SD for each plate are presented below the typical growth pattern distribution seen in the particle release. In all the releases the sampling was run for 5 minutes at 28 l/min resulting in the potential total capture of 3000, 300 and 30 CFU respectively. (B) SEM (left) and fluorescent microscopy (right) of *M*. *smegmatis*::*gfp* isolated on a PM10 impactor following experimental release.

In order to confirm the ability to capture live mycobacteria in the RASC, a Six-Stage Viable Andersen Cascade Impactor was tested in a series of 3 replicates of 3 wet *M*. *smegmatis*::*gfp* test releases (Fig 4). These experiments incorporated a range of cell concentrations—from ~0.2 CFU/L to ~22 CFU/L of RASC air—to ensure the capacity of the RASC to capture the variable numbers of *Mtb* bacilli expected to be produced by TB patients. The Andersen impactor comprised six stages, with different aerodynamic particle sizes captured on the solid 7H10 agar plate contained in each stage. The first plate (A1) collected the largest particles (>8 μm) and the last plate (A6) collected the smallest particles (< 1 μm). Each of the other plates had a collection size range between the two extremes. At the highest concentration range, the colonies growing under the jets of the Andersen device were too numerous to count individually with accuracy. In the bottom row, for the extremely dilute sample, a mean of 15 colonies were captured in the impactor giving a capture efficiency of 49.3% of expected CFU. As the protocol evolves, and more is known about the patient output, the sampling times for the impactor can be adjusted to be less sensitive (shorter sampling times) or more sensitive (longer sampling times). Based on preliminary data, we expect that an active TB patient will generally produce < 1 CFU/L, which lies between the last two rows in Fig 4A. After each release, we swabbed a 15x15 cm square with a sterile felt filter moistened with sterile PBS solution on three walls of the chamber (just above the release point, on the wall opposite the release point and on a side wall) and we did not grow any colonies from any of the releases indicating that there was practically no adhesion of viable aerosol to walls of the chamber.

### Imaging bacilli captured after aerosolization

To study the morphology of particles (including bacteria) isolated in the RASC, samples were captured onto metal plates in a three-stage Dekati impactor. The utility of this system was validated following release of *M*. *smegmatis*::*gfp*: a representative image (Fig 4B, left panel) presents a 20 μm × 20 μm view of a metal foil inserted on the final Dekati impactor plate which collects particles in the 2.5 μm to 1 μm range. A single *M*. *smegmatis*::*gfp* bacillus is visible near the center of the image. The identity of the *M*. *smegmatis* test strain was confirmed in the right-hand panel, containing a 20 μm × 20 μm fluorescent photomicrograph of the same sample, in which the bacillus was brightly fluorescent. It was noticeable, though, that this particular organism appeared to exhibit a slightly less curved morphology than that observed in the SEM image, perhaps as a consequence of the fluorescence signal.

### Demonstrating the utility of the RASC in a TB clinic

In order to demonstrate key capabilities of the RASC, a standard protocol was developed that was approved by the University of Cape Town Faculty of Health Sciences Human Research Ethics Committee. Participants provided written informed consent prior to acceptance into the study.

Adult patients with a recent diagnosis of TB were recruited immediately prior to initiation of therapy. After entering the RASC, the sealed door was closed; the surgical mask removed, and a cough sputum sample provided. Following occupation of the RASC, carbon dioxide levels increased consistently, reaching levels of approximately 4,000 parts per million (ppm) above ambient levels owing to exhaled breath. Given that the concentration of CO_2_ in exhaled air is close to 40,000 ppm, the target concentration of 4,000 ppm above ambient atmospheric level (typically ~400 ppm) indicated that approximately 10% of the air in RASC comprised expired air at that point. The typical time required for CO_2_ levels to reach the target concentration was approximately 25 to 30 min. During this period, only the APS was set to sample from the chamber, at a rate of 5 l/min. Once the CO_2_ level reached the 4,000 ppm target, sampling was initiated at approximately 50 l/min: the first 10 minutes were utilized for collection of bacteriology samples (Andersen impactor and gel filter), after which a further 10 minutes was allowed for imaging and molecular samples (PM10 impactor and polycarbonate filter). This 20 minute sampling period was expected to maintain CO_2_ levels at approximately the target concentration. Finally, high flow sampling was initiated at 300 l/min for PCR samples, a 10 minute process in which the CO_2_ concentration was expected to return to background ambient levels. Details of the flows out of the RASC are given in Table 1. The total sample therefore represented the expired air over the full study period of approximately 60 minutes. The mathematical relationship between CO_2_ production, resultant CO_2_ levels, and effect of sampling rates, is described in Appendix 1 (S1 File).

**Table 1:** Sampling rates of the capture devices and the periods in which they are used.

Sampling period	Sampling Device	Flow out of booth	Total outflow
Bacteriology sampling	Andersen Impactor	28 l/min	
	Gelatine Filter	20 l/min	
	Aerodynamic Particle Sizer	5 l/min	53 l/min
Imaging Sampling	PM10 Impactor	30 l/min	
	Polycarbonate Filter	20 l/min	
	Aerodynamic Particle Sizer	5 l/min	55 l/min
PCR sampling	Felt Filter	Approx. 300 l/min	
	Aerodynamic Particle Sizer	5 l/min	Approx. 300 l/min

The air being removed from the sampling space is replaced through large HEPA filters. The laser scattering particle counter and CO_2_, temperature and humidity monitor run at low flow rates and the exhaust air is returned to the sampling space. During the patient sampling the liquid impingers were not used.

**Fig 5:**
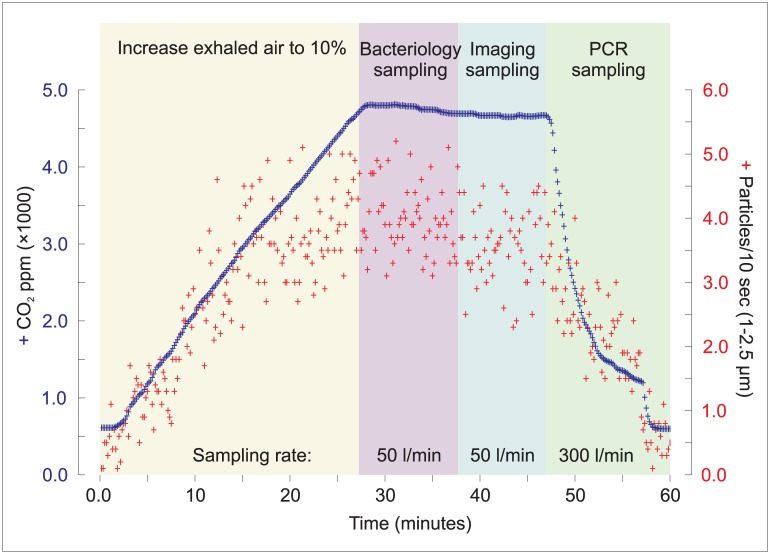
Particle production as a function of respiration in a clinical TB patient. CO_2_ concentration (solid line and left ordinate) and particle counts (dots and right ordinate) in the 1–2.5 μm size range for a TB patient.

During the entire course of the 60 minute protocol, aerodynamic particle size was determined at 10 second intervals along with continuous monitoring of CO_2_, humidity and temperature levels. Fig 5 details RASC CO_2_ concentrations and particle size counts (in the 1–2.5 μm size range) for a single patient. It is notable that changes in particle count parallel the CO_2_ concentrations within the RASC: as the patient exhaled and coughed over the first non-sampling 30 min period, the CO_2_ and particle count built up; subsequently, the counts plateaued, ultimately reaching an approximate steady state as the samplers evacuated the sample space; and, finally, the counts dropped rapidly as the chamber was exhausted with a high-volume sampler. From a preliminary analysis of data collected, the temperature increased by 5.9 degrees during the whole sampling period (24.0 to 29.9°C, n = 19) and the humidity increased by 10.9% from the start of the sampling to the bacterial and imaging sampling and then reduced by 11.9% during the PCR sampling (52.7% through 63.6% to 51.7%, n = 19).

**Fig 6:**
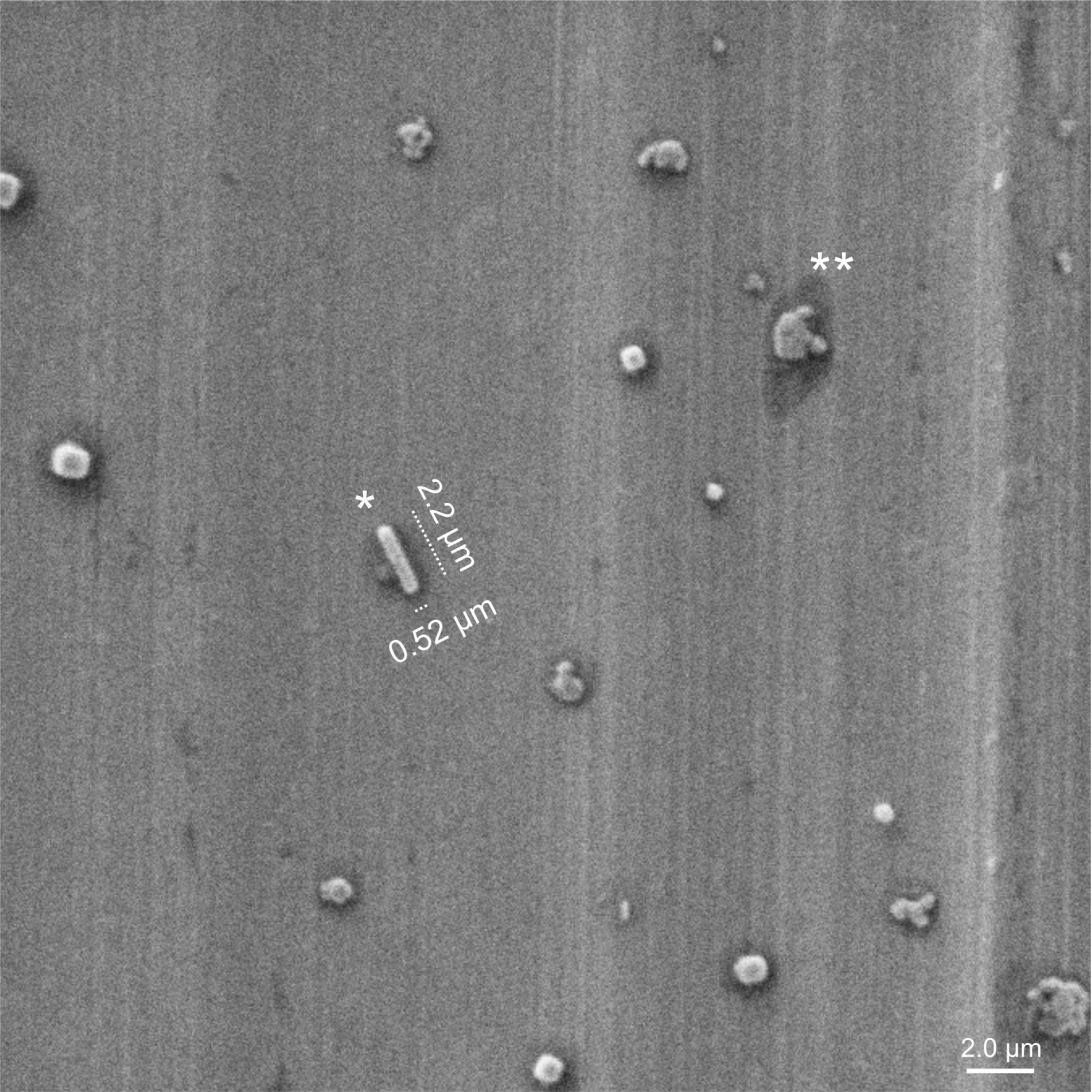
Isolation of *Mtb* from a TB patient. SEM image of patient sample impacted on the lower plate of the PM10 impactor. The dimensions and morphology of the rod-shaped structure (denoted by *) are consistent with the presence of *Mtb* bacilli in the untreated TB patient. There is also evidence of multiple “splats” of unknown identity (one example is denoted by **) which might comprise organic matter derived from patient lung or respiratory tract. Note the “halo” structures (dark shadows) surrounding each particle.

It is evident that the aerosol particle data (such as those shown in Fig 5) will become most important when correlations between particle counts and disease status can be drawn by comparisons across multiple patients. The preliminary patient samples are too few to enable any definitive inferences; nevertheless, these early results enabled a key observation from the very first patient tested in the RASC: a single putative *Mtb* bacillus is visible in an SEM image obtained under high magnification of a 10 μm by 10 μm region of the lower plate of the Dekati sampler (Fig 6). In the full image, multiple similar rod-like organisms were clearly visible (data not shown). The presence of *Mtb* in the chamber was confirmed by culture of a colony isolated on plate 5 of the Andersen impactor, and preliminary genotyping through PCR amplification of the RD9 locus which differentiates *Mtb* and *M*. *canettii* from other mycobacteria within the *Mycobacterium tuberculosis* Complex [16].

A number of other particles were visible on the Dekati plate (Fig 6), and these likely arose from the respired air from the patient, especially since the particulate background in the chamber was quite low. Most of these particles were characterized by a surrounding “halo”, an observation which suggested the intriguing possibility that this feature was caused by dried liquid that might contain complementary biomarkers of *Mtb* infection. Again, however, more samples—from bothTB patients and control, non-TB active individuals—will need to be processed before a definitive correlation can be dawn between halo formation (and composition) and TB disease status.

## Discussion

*Mtb* survival during the airborne phase of its life cycle is an understudied area of research. In contrast to prior studies which have focused on culture of aerosols created immediately following periods of forced coughing [17,18], the RASC enables study of more aged airborne organisms that have been selected or adapted for environmental survival.

The RASC was developed to enable quantitation and characterization of the bio-aerosols emitted during normal breathing and coughing of TB patients in future clinical studies, and to incorporate the resulting information into a refined model of TB transmission. Critically, this long-term goal requires that the performance levels of the sampling sub-systems within the RASC be calibrated to ensure reproducibly robust bio-aerosol detection, quantification, and isolation. The results presented here suggest that the RASC is performing as intended and can be usefully applied in collecting and characterizing patient-generated aerosols, and for assessing changes in the nature and content of respired aerosols over time. The air mixing in the RASC is designed to replicate air currents that occur in normal indoor environments, and which decrease particle settling compared to still air. Information that can be acquired in the RASC therefore includes the number, size distribution (physical and aerodynamic), and morphology of aerosolized particles.

Infection of a new human host by *Mtb* depends on the generation of a source aerosol population that is defined both by the number of bacilli and the phenotypic and genomic characteristics of the organisms that are emitted from the infected individual into the environment. In addition, it is important to consider the evolution of the emitted particles as a function of particle size and morphological structure since these parameters alter how they travel and are transformed in the environment [19]. Particularly important in this context are the processes by which the aerosol particles dry (with environmentally variable kinetics) into derivative (smaller) particles of size and morphology that have been shown to promote travel deeper into alveolar spaces in the lungs of the recipient [20].

We did demonstrate the novel ability to image individual airborne rod shaped organisms. However, we are currently unable to definitely identify these as Mycobacteria or other contaminating organisms. The general morphology of individual mycobacteria is a rod shape, approximately 0.2–0.5μm in cross-sectional diameter and 1–4μm in length [21]. However, bacillary dimensions are known to vary according to whether observed in sputum and bronchoalveolar lavage (BAL) fluid or in TB cavities: organisms found in sputum and BAL are significantly longer, whereas the lengths of bacilli harvested in cavities are more reminiscent of stationary phase bacilli *in vitro* [22]. Under certain growth conditions, colonies can also appear in groups as elongated “cord” structures [23]. It seems plausible, therefore, that some proportion of the emitted aerosol produced by the infected donor may retain the elongated features of the microorganisms in the airways depending on the composition of the airway fluid in which they are ejected. This is also likely to be a function of the region of the lungs of origin, as it is known that the periphery is largely composed of surfactant whereas the ciliated airways possess mucus containing glycolipids and glycoproteins. The presence of inflammatory cells, immune mediators, and general debris associated with infection almost certainly contributes to a complex scenario which cannot easily be predicted and requires direct experimental evidence.

Once in air, drying of the aerosol will proceed at a rate dictated by ambient temperature and humidity (Appendix 2, S2 File). The impact of exposure for individuals breathing nearby in either residential or occupational settings will therefore vary in both space and time, and must be heavily influenced by particle morphology at the time of inhalation. With regard to the influence of other key single particle features such as shape and agglomerate density, there are known phenotypes that might promote a rod shaped presentation (referred to as “cording” in Koch’s original description of *Mtb* [24]) that would further enhance the tendency for peripheral lung deposition [25].

Our preliminary results suggest that RASC sampling has the potential to give qualitative information about particles and organisms which have been exhaled and remained airborne. A standardized protocol, together with the ability to monitor the proportion of sampled air as a function of total exhaled air, should enable quantitative assessments of particle and organism production rates from infected individuals. Importantly, the potential to acquire quantitative data suggests the additional opportunity for inter-personal variation to be assessed and for intra-personal variability to be determined in repeated investigations, including after initiation of treatment.

Transmission within TB endemic populations can be modeled [26] from the measured volume of air rebreathed from others which is determined by socio-environmental factors [27,28], the proportion of rebreathed air derived from prevalent TB infected individuals in that population, and the surviving pathogen concentration of exhaled air from an infected individual [5]. Quantitative assessments of pathogen load in exhaled air may allow more accurate modeling of those individuals and factors which are driving the elevated rates of TB disease which characterize high-burden settings such as our own [29].

## Supporting Information

S1 File Appendix 1 describing the mathematical relationship between CO_2_ production, resultant CO_2_ levels, and effect of sampling rates in the RASC. (DOCX)

S2 File Appendix 2 describing the drying of the aerosol at a rate dictated by ambient temperature and humidity. (DOCX)
