# Supplementary material for: Real-Time Investigation of Tuberculosis Transmission: Developing the Respiratory Aerosol Sampling Chamber (RASC)
Source: PLoS One. 2016 Jan 25;11(1):e0146658. doi: 10.1371/journal.pone.0146658 (PMC4726558; doi:10.1371/journal.pone.0146658)
Supplement: S1 File — (DOCX) [file pone.0146658.s001.docx]

# Appendix 1

## CO_2_ Measurements

Carbon dioxide (CO_2_) levels within the RASC are monitored continuously. CO_2_ is a natural tracer gas produced during normal respiration that is used to quantify the volume of expired air that has been sampled. The concentration of CO_2_ in exhaled air is close to 40,000 parts per million (ppm) so that a concentration of 4000 ppm above ambient atmospheric levels indicates that 10% of the air in RASC is expired air. The mathematical relationship between CO_2_ concentration, CO_2_ production, and CO_2_ clearance is shown below.

Mathematically, accumulated exhaled air in the RASC of volume V, is equal to the rate of exhaled air generated by a participant (*npCa*) plus ambient air in the RASC (*QCe*) minus indoor air removed by sampling (*QCi*):

$$V\frac{dC_{i}}{dt}=npC_{a}+Q\left( C_{e}-C_{i} \right)$$

where *C_i_* denotes RASC carbon dioxide concentration (ppm), *Q* is the sampling rate (L/s), *C_e_* is the outdoor carbon dioxide concentration (ppm), *p* is the breathing rate of the person in the RASC (L/s), *C_a_* is the carbon dioxide fraction contained in breathed air and *n*=1 is a single participant in the RASC.

Rearranging (1) and integrating with respect to time from CO_2_ = *C_o_* when time, t = 0 to CO_2_ = *C_T_* when time, t = T, we obtain the sampled exhaled air expression that describes the concentration-time curves:

$$C_{\left( T \right)}=C_{e}+\frac{npC_{a}}{Q}\left[ 1-e^{\frac{-QT}{V}} \right]+\left( C_{0}-C_{e} \right)\left[ e^{\frac{-QT}{V}} \right]$$

where *C_o_* is the RASC carbon dioxide at time, t = 0, which is greater than *C_e_*.

Examining (2) indicates that when the sampled exhaled air is exhausted from the RASC at a high flow rate such that *npC_a_* << *Q*, the equation simplifies to:

$$C_{\left( T \right)}=C_{e}+\left( C_{0}-C_{e} \right)\left[ e^{\frac{-QT}{V}} \right]$$

and produces the exponential decay curve observed during high flow rate sampling.
