# Supplementary material for: Real-Time Investigation of Tuberculosis Transmission: Developing the Respiratory Aerosol Sampling Chamber (RASC)
Source: PLoS One. 2016 Jan 25;11(1):e0146658. doi: 10.1371/journal.pone.0146658 (PMC4726558; doi:10.1371/journal.pone.0146658)
Supplement: S2 File — (DOCX) [file pone.0146658.s002.docx]

# Appendix 2

**Aerosol dynamics**

It is known that, upon inhalation by the recipient, the major factor governing lung deposition is particle size, shape, and distribution. Aerodynamic size encompasses the effects of density and shape as described in Stokes’ equation as follows [18]:

$$V_{T}=D_{a}^{2}\frac{\left( \rho_{0}C\left( D_{a} \right)g \right)}{\text{18}}=D_{v}^{2}\frac{{(\rho}_{p}C\left( D_{v} \right)g)}{\left( 18 \right)}$$

where *V_T_* is the terminal settling velocity; *g* is the acceleration due to gravity; *η* is the viscosity of the surrounding fluid (air); *χ* is the dynamic shape factor; *ρ_0_* and *ρ_p_* are unit (1g/mL) and true particle density, respectively; *D_a_* and *D_v_* are equivalent aerodynamic and geometric (volume) diameters; and *C(D_a_)* and *C(D_v_)* are slip correction factors that are applied to each particle size when below 1μm to account for the discontinuity of the surrounding medium as the particle approaches the mean free path of the gas.

Particles less than 5μm in aerodynamic diameter can enter the upper airways, with a proportion reaching the lungs readily [19]. This is the first requirement for transmission to occur successfully. Assuming this requirement is met, then secondary phenomena may also become important. Rearranging the above expression to solve for aerodynamic diameter helps identify the potential significance of these secondary effects.

$$D_{a}=D_{v}\left( \frac{\rho_{p}C\left( D \right)}{} \right)^{\frac{1}{2}}$$

where *C(D)* is a composite slip correction factor $\left[ C\left( D_{v} \right)/C\left( D_{a} \right) \right]^{\frac{1}{2}}.$

This expression indicates that, where the dynamic shape factor is greater than 1 (that is, where the cross sectional area diameter is smaller than the length), it will behave as an aerodynamically smaller particle than it appears geometrically. Also, where the density of the particle is less than 1, the particle will behave as an aerodynamically smaller particle than it appears geometrically. In addition, if the particle is smaller than 1μm, it will ‘slip’ between gas molecules and behave as a smaller particle. The latter is of importance for *Mtb* with rods that may be very small in two dimensions and, statistically, align with flow. As an example, the aerodynamic diameter could be half of the geometric diameter for the rod-shaped particles with length/diameter ratio of 4 owing to the shape alone.
